# If it doesn’t help, it doesn’t hurt? Information elaboration harms the performance of gender-diverse teams when attributions of competence are inaccurate

**DOI:** 10.1371/journal.pone.0201180

**Published:** 2018-07-19

**Authors:** Hans van Dijk, Bertolt Meyer, Marloes van Engen

**Affiliations:** 1 Department of Organization Studies, Tilburg University, Tilburg, The Netherlands; 2 Department of Organizational- and Economic Psychology, Technische Universität Chemnitz, Chemnitz, Germany; 3 Department of Human Resource Studies, Tilburg University, Tilburg, The Netherlands; Indiana University, UNITED STATES

## Abstract

Information elaboration—the act of exchanging, discussing, and integrating information and perspectives through verbal communication—tends to be considered as the silver bullet that drives the performance of diverse teams. We challenge this notion by proposing that the effect of information elaboration on team performance depends on the accuracy of within-group competence attributions, i.e. the extent to which attributions of task competence among team members correspond with members’ actual task competence. We argue that information elaboration may actually harm performance when within-group competence attributions are inaccurate, given that in such teams decisions are likely to be based on suggestions from members who have much influence but little competence. We conducted an experiment with 97 gender-heterogeneous teams working on gender-typical problems and coded their interactions. Our findings support our hypotheses that members who are perceived as more competent are more influential in the information elaboration process, and that information elaboration harms performance when competence attributions are not accurate. In contrast to our expectations, pro-diversity beliefs did not mitigate this negative effect of inaccurate competence attributions. We argue that this speaks to the robustness of our findings regarding the detrimental effects of information elaboration when competence attributions are inaccurate.

## Introduction

Research on the consequences of diverse individuals working together to accomplish a task has become increasingly prominent over the past decades as societies have become more diverse and work has increasingly been structured around teams [1]. Although research on diversity in teams has yielded many inconclusive findings (e.g., [2, 3, 4, 5, 6]), it has become an accepted notion that information elaboration is key to the performance of diverse teams [7, 8, 9, 10, 11, 12]. Information elaboration refers to the act of exchanging, discussing, and integrating information and perspectives through verbal communication [9, 11]. This process of information elaboration is considered to enable diverse teams to leverage on the richer pool of information, perspectives and skills that they are believed to have at their disposal.

Two recent reviews of the diversity literature [13, 14] however suggest that there may be a benefit to diversity in teams that does not require teams to engage in information elaboration, namely that diversity facilitates coordination via the recognition of task-specific competence. Differences in functional background can, for example, serve as a cue regarding members’ unique expertise and competencies, and differences in age and tenure tend to be cues of experience [15, 16]. Compared to more homogeneous teams where members lack cues for competence, differences between team members thus can more easily lead to a distribution in tasks and roles and facilitate an understanding of whom to turn and listen to in case questions and problems emerge in a specific task domain. However, an important caveat of this potential coordination advantage of diversity is that its benefit depends on the extent to which the attributions of expertise and competence are *accurate*, i.e. correspond with a target member’s actual competence on a task. If they are inaccurate, team members may erroneously attribute high levels of competence to the wrong person and thus put their trust in a less competent team member [14].

In this article, we examine the implications of this potential coordination advantage of diversity for the relationship between information elaboration and team performance in gender diverse teams. Building on research to the role of attributions of competence (e.g., [17, 18]) and status (e.g., [19, 20]) in teams, we argue that team members during information elaboration rely more on members who are perceived as more competent [19, 21]. The reason for this is that teams mainly engage in information elaboration when they experience uncertainty, and uncertainty causes individuals to seek for and rely on cues regarding expertise and competence. We argue that the resulting disparity in members’ influence on the information elaboration process may benefit performance when those members who are perceived as more competent also *are* the most competent team members, but that it hurts performance when attributions of competence are inaccurate and the most influential team members thus are those who are less competent. As such, we assert that when attributions of competence in a team are inaccurate, information elaboration can actually harm team performance. We focus specifically on gender diversity because it is omnipresent and known to affect attributions of competence on many—if not most—tasks in organizations [22, 23].

To examine how this potentially negative consequence of inaccurate attributions of competence can be attenuated, we designed a study that manipulated members’ diversity beliefs, i.e. the “beliefs about the value of diversity to work group functioning” [24]. Because pro-diversity beliefs stimulate team members to have more consideration and regard for other team members, we propose that they may increase the extent to which the input of members who are initially perceived as less competent will be requested and valued. In addition, pro-diversity beliefs may spur such low-status team members to feel less anxious and hence more empowered to voice their opinion (cf. [25]). We thus argue and examine to what extent pro-diversity beliefs reduce the extent to which information elaboration hurts performance when competence attributions are inaccurate.

We advance theory and research on the role of information elaboration in (gender) diverse teams (e.g., [11; 26]) by arguing and showing that some members are more influential in the process of information elaboration than others, that these disparities in influence are related to team performance in such a way that information elaboration can actually harm performance when less competent team members are relatively influential, and by examining whether diversity beliefs mitigate such a negative effect of information elaboration on performance. We examined these relationships in an experiment involving 97 four-person gender-diverse teams working on a team task. A particularly strong point of our study is that we used objective measures for team performance and behavioral observations to measure influence and information elaboration, which enabled us to shed a light at ongoing processes in gender diverse teams and thereby substantiate our claims.

### Information elaboration in diverse teams

Diversity refers to “differences between individuals on any attribute that may lead to the perception that another person is different from self” [11]. In examining the relationship between team diversity and team performance, diversity researchers have typically examined to what extent differences among team members’ characteristics, such as gender, age, functional background, personality, and values, affect team performance. Over 50 years of research has led to the conclusion that diversity is consequential, but that its effects remain to be fully understood [1, 27, 6].

Part of the confusion stems from the fact that different processes are at play in diverse teams that account for different outcomes. The negative consequences of diversity are generally attributed to social categorization processes [28, 29] and similarity/attraction [30]. According to the social categorization perspective, people categorize others into ingroup and outgroup members based on the extent to which they are perceived as similar or different, respectively. This categorization is consequential as people have been found to respond more favorably toward ingroup members than toward outgroup members in a variety of ways, including the level of affection, the willingness to cooperate, and the level of trust [31, 32]. Based on the social categorization perspective it can therefore be expected that the group processes in homogeneous teams are more cooperative and hence productive than the group processes in more diverse teams [11]. In line with these assumptions, several studies have shown that perceptions of within-team diversity lead to decreased levels of team social integration, interaction, and performance (e.g., [33, 34, 9, 35, 36]).

In contrast, the information/decision-making perspective [11, 37] suggests that diversity should be conceptualized as an informational resource: differences between the team members are expected to reflect a richer and more heterogeneous pool of insights, knowledge, and perspectives. Grounded in a conceptualization of groups as information processing systems [38], this richer pool of task-relevant information is believed to enable diverse teams to reach higher quality decisions and solutions than more homogeneous work teams when they are able to integrate the pool of task-relevant information. Information elaboration is thought to be the key process that drives this integration, thus enabling diverse teams to live up to their potential and outperform homogeneous groups.

Information elaboration in teams has been defined in different ways. Van Knippenberg et al. [11] defined it as “the exchange of information and perspectives, individual-level processing of the information and perspectives, the process of feeding back the results of this individual-level processing into the group, and discussion and integration of its implications”. However, others noted that this definition is overly broad, because it mixes interindividual behavioral communication processes with intraindividual cognitive processing [9]. Research on the hidden profile paradigm (e.g., [39]) has demonstrated that sharing and discussing information on the one hand, and individual-level use and processing of that information on the other hand, are different things. To avoid this conceptual ambiguity, we define information elaboration in the same way as Meyer et al. [9], i.e. “the act of exchanging, discussing, and integrating information and perspectives through verbal communication”. Additional advantages of this definition are that it focuses on the aspect that is generally understood to be key to team functioning (i.e. information sharing, discussing, and integrating; [38]) and that is measurable [40]. It is also more in line with how information elaboration is commonly measured, i.e. by observing and coding group discussions and thus focussing on the behavioral component (e.g., [8, 9, 10]).

Although limited in number, the studies that have examined the effect of information elaboration on the performance of diverse teams support the notion that information elaboration enhances performance (e.g., [8, 41, 10]). Two recent studies qualified the extent to which information elaboration represents a silver bullet for diverse teams by showing that information elaboration does not enhance performance in all task contexts. The first study showed that information elaboration only enhances the performance of diverse teams in turbulent, but not in stable environments because in such environments teams function more based on routines [26]. The second study [12] showed that information elaboration positively relates to performance when members hold different pieces of task-relevant information, but that information elaboration does not affect performance when all members hold the same pieces of information. Whereas these studies are the first to assess under what conditions information elaboration does not enhance performance in diverse teams, the theoretical arguments do not diverge from the initial theory on the value of information elaboration in diverse teams and its boundary conditions [11]. As a consequence, the current state of the science still suggests that information elaboration in general enhances performance in diverse teams, and that when it does not help, it does not hurt either.

However, based on recent theoretical developments in research on diversity in teams, we contend that this is a premature conclusion. Two recent reviews of the literature on diversity in teams have argued that diversity can yield coordination benefits that enable diverse teams to outperform homogeneous teams without the need to engage in information elaboration [13, 14]. Specifically, this coordination perspective on diverse teams entails that differences between team members serve a signalling function that facilitates coordination in two ways. The *first* is that differences between members tend to serve as a cue for members to expect divergent ideas and perspectives, which also makes them more open for and ready to accept differences in throughts and opinions [42, 13]. The *second* is that a member’s characteristics signal a member’s expertise (e.g., university degree in chemistry) and experience (e.g., age), which is vital information in team work for knowing whom to trust, delegate tasks to, listen to, and ask for advice [43, 44, 14]. Taken together, these signalling functions of diversity suggest that in diverse teams, members tend to use the differences in characteristics between members for attributing competence and rely on those attributions for accepting other members’ suggestions and judgments. This aligns well with role congruity theory [22], which suggests that in particular gender is used in organizations to assign roles and tasks to individuals: The more that a gender role (e.g., woman) and corresponding attributions of competencies (e.g., nurturing) are congruent with the role (e.g., caregiver) and required competencies for a particular task (e.g., care giving), the more likely it is that an individual will be perceived as competent.

Van Dijk et al. [14] have argued (but not shown) that this can be beneficial for the team when such attributions of competence are congruent with members’ actual competence, but can harm performance when attributions of competence are inaccurate. We build on that argument by applying it to theory on information elaboration and asserting that such inaccurate attributions of competence are particularly detrimental when (diverse) teams engage in information elaboration. We base this assertion on two complementary arguments regarding the process of information elaboration. The first is that information elaboration in diverse teams is likely to be a disparate process in which members tend to be more influential to the extent that they are perceived as more competent. The second is that more influential members have a stronger bearing on the performance of a team, and that information elaboration thus enhances performance when competence attributions are accurate but harm performance when competence attributions are inaccurate. In the following we further specify each argument.

### Disparate information processing due to attributions of competence

There is limited theory on the process of information elaboration, meaning that we do not exactly know *how* information elaboration improves the functioning of teams (cf. [26]). However, one core assumption that seems to underlie theory on information elaboration is that information elaboration yields the best outcomes when all members are equally involved in and contribute equally to the information elaboration process. This assumption rests on the idea that disparities in the information elaboration process result from social categorization and, more specifically, intergroup bias [11]. Intergroup bias causes members to trust information that is shared by ingroup members more than information that is shared by outgroup members [45]. The presence of outgroup members in diverse teams thus increases the chance that team members do not pay attention to and elaborate upon task-relevant information [46]. Accordingly, van Knippenberg et al. [11] proposed that “intergroup biases elicited by work-group diversity are disruptive to elaboration of task-relevant information and therefore to group performance” (cf. [9]).

Intergroup bias is however not the only reason for disparities in the information elaboration process to emerge. From a status perspective [47, 48, 21], disparities in the information elaboration process are likely to be present in any team where members differ in the extent to which they are perceived as competent by their fellow team members. For example, expectation states theory [19] posits that team members make competence attributions in order to adjust their behavior based on their expectations of fellow team members’ level of task competence. The aim of such assessments of fellow team members’ task competence thus is to understand what use or value fellow team members have to the perceiver [49, 17]. When team members are attributed high levels of task competence, the perceiver believes that such team members are likely to provide valuable contributions to the team. As a consequence, perceivers are more likely to defer to such team members and give them opportunities to participate, thereby granting more influence to team members who are perceived as more competent [50, 43, 51, 18].

One implication of this tendency of teams to grant more influence to members who are perceived as more competent is that information elaboration is not an equal process where information flows evenly among team members. Indeed, prior research indicates that discussions in teams can be characterized as highly disparate processes where members who are perceived as more competent (in comparison to members who are perceived as less competent) share more information [52, 53, 54], share more unique information [55, 56], and where information that is shared by members who are perceived as more competent is remembered and elaborated more than information that is shared by members who are perceived as less competent [57, 58]. Especially this latter finding demands attention, given that it suggests that judgments about the merit of a contribution is weighed based on the extent to which a member is perceived as competent at the task at hand [59]. Attributions of competence thus serve as an implicit coordination mechanism to flesh out which contributions (or, more specifically, *whose* contributions) are most likely to benefit the decision-making process (cf. [14]).

We argue that team members are particularly likely to rely on such attributions of competence during information elaboration processes, given that members tend to engage in information elaboration under conditions of uncertainty. There is little use in exchanging and discussing information when there is agreement and clarity on what needs to be done. However, when there is uncertainty members do need to engage in information elaboration, either to collectively come to terms with an issue at hand, or to clarify divergent ideas and perspectives among team members. We argue that under such circumstances, members feel the need to rely on their attributions of competence in order to make sense of all information and perspectives that are shared and distinguish between more versus less valuable contributions. Accordingly, we hypothesize that information elaboration is likely to be a disparate process where members who are perceived to be more competent are more influential than members who are attributed low levels of competence.

*Hypothesis 1*: *Members who are perceived as more competent are more influential in the information elaboration process compared to members who are perceived as less competent*.

### The accuracy of competence attributions, information elaboration, and team performance

Because attributions of competence are nothing but perceptions based on arbitrary cues, there is a reasonable chance for such attributions to be inaccurate [20, 49]. In a gender diverse team, for example, higher levels of competence tend to be attributed to men compared to women on masculine tasks given that prevalent stereotypes suggest men to be more competent in a masculine task context [60, 61]. These stereotype-based attributions however do not indicate whether or not men team members actually are better at such tasks than women team members, which entails that the most influential team members may not be the most competent team members.

Whereas a number of studies suggest that such inaccuracies in competence attributions will be overcome when members work together and interact over time (e.g., [20, 62]), other studies suggest that such inaccuracies are likely to remain and have a lasting effect on team functioning and performance given that status and influence tends to maintain and reinforce itself, such that those who have higher levels at the outset of a task tend to have more–or at least an equal amount of—influence at the end of the task [51, 63]. In the absence of clear feedback, we argue that this self-reinforcing mechanism of influence is likely to be the same during information elaboration, given that information that is shared by members who are perceived as more competent tends to be valued more than information that is shared by members who are perceived as less competent [57, 58]. The influence of members who are perceived as more competent is thus likely to remain–and potentially even increase—over time.

We argue that this is beneficial in teams where competence is attributed more accurately. In such teams, the expertise of more competent team members will be utilized the more that information elaboration takes place. We therefore expect that in teams with more accurate competence attributions, information elaboration enhances team performance. However, based on the same line of reasoning we expect the opposite for teams with more inaccurate attributions. In such teams, members’ trust in the contributions of members who are perceived as more competent will be misplaced. We therefore expect that in teams with more inaccurate competence attributions, the disparately high influence of members who are inaccurately perceived as more competent is likely to inhibit team performance as the information elaboration process unfolds over time.

*Hypothesis 2*: *The effect of information elaboration on team performance is moderated by the extent to which attributions of competence within a team are accurate*, *such that information elaboration enhances performance when attributions of competence are more accurate*, *but diminishes performance when attributions of competence are more inaccurate*.

In concert, Hypotheses 1 and 2 advance information elaboration theory by arguing that (a) disparities in the information elaboration process can enhance the extent to which information elaboration enhances performance (under conditions of more accurate competence attributions), and (b) information elaboration can hurt performance (under conditions of more inaccurate competence attributions). Our final question concerns the extent to which the expected negative consequences of inaccurate competence attributions can be attenuated. In the following, we propose that pro-diversity beliefs may do just that.

### Inhibiting the negative consequences of inaccurate competence attributions through diversity beliefs

Research on diversity beliefs traditionally distinguishes between pro-diversity beliefs, i.e. the belief that diversity is valuable to team functioning, and pro-similarity beliefs, i.e. the belief that diversity is disruptive to team funcitoning. Pro-diversity beliefs have been shown to enhance the performance of diverse teams [8], which is considered to be due to the enabling effect of pro-diversity beliefs on information elaboration in diverse teams [35]. Indeed, from a social categorization perspective, pro-diversity beliefs are likely to make ingroup members more receptive to the input from outgroup members, thus removing the barriers between subgroups that inhibit information elaboration.

We are interested in whether or not pro-diversity beliefs have a similar effect on the proposed disparity in information elaboration by shaping members’ receptivity for input provided by members who are perceived to be less competent. Specifically, we argue that pro-diversity beliefs may cue members not to judge a contribution based on the person who makes the contribution [51, 59], but on the content or merit of the contribution itself. In a similar way as pro-diversity beliefs thus motivate members to not discard input from outgroup members based on intergroup biases, pro-diversity beliefs then are expected to motivate members not to discard input from members who are perceived as less competent.

Whereas we expect that pro-diversity beliefs attenuate the extent to which information elaboration in teams with more inaccurate competence attributions hurts performance, we do not expect that they affect the extent to which information elaboration in teams with more accurate competence attributions increases performance. Pro-diversity beliefs may stimulate members’ receptivity for input from members who are perceived as less competent given that they point at the potential value of members whose competence may be underestimated. Pro-diversity beliefs however do not call for more scrutiny regarding the input from members who are perceived as more competent. We thus do not expect that pro-diversity beliefs will affect team members’ attitudes towards the contributions of members who are perceived as more competent. Accordingly, we hypothesize that pro-diversity beliefs foster the extent to which members are receptive to input provided by members who are perceived as less competent, and therefore reduces the extent to which information elaboration harms performance in teams with less accurate competence attributions:

*Hypothesis 3*: *Diversity beliefs attenuate the negative effect of inaccurate competence attributions on the relationship between information elaboration and performance*, *such that information elaboration in teams with more inaccurate competence attributions is less detrimental under the condition of pro-diversity beliefs compared to pro-similarity beliefs*.

## Method

Our sample consisted of 97 four-person gender-diverse teams comprising 186 men and 202 women with an average age of 26.88 years (*SD* = 7.93). There were 28 teams with one woman and three men, 36 teams with one man and three women, and 33 teams with two women and two men. The teams worked either on math or on emotional intelligence (EI) problems. Because men are stereotypically believed to be more competent in math compared to women, but women are stereotypically believed to be more competent in EI compared to men, we used these two different task settings to (a) create disparities in competence attributions among team members while (b) ruling out the possibility that gender effects may affect or explain our findings (see [50] for a similar design).

To establish the validity of the task-gender stereotype, we conducted a pre-test with a different sample: We randomly assigned 107 participants (73 female, mean age = 27.70 years, SD = 9.46) to work individually on either ten math items or on ten EI items. Afterwards, participants assessed how well men in general perform in this task and how well women in general perform on this particular type of task. A mixed within-and-between ANOVA that included task type and participant gender as between-participant factors and the expeced performance of men and women as within-participant repeated measures revealed a performance expectation × task type interaction, *F*(1,205) = 53.51, *p* < .001, η^2^ = 0.20: On average, participants rated the expected performance of men for the math task at 3.67 (*SD* = 0.61) and for the emotional intelligence task at 3.00 (*SD* = 0.70). Women’s performance was expected to be 3.05 (*SD* = 0.65) for the math task and 3.68 (*SD* = 0.59) for the EI task. Thus, in support of the assumed stereotypicality of the task and regardless of their own gender, participants expected a higher performance from women working on emotional tasks compared to math tasks, while for men this pattern was reversed.

The study was advertised as a voluntary study on how people work together in teams at the department of psychology at the University of Zurich and on an e-mail listserv operated by the department where students could opt in to receive announcements about studies conducted at the department that seek participants for payment. We offered an incentive of 1.5 experimental participation credits (psychology students in Zurich need to collect 20 of these credits during their undergraduate studies) or an alternative monetary compensation of 25 Swiss Francs (approximately 25 USD) for participation. Participation in the study was voluntary. We assured study participants verbally and in writing that all data would be collected anonymously and that it would be treated confidentially such that no individual participants can be identified. The experimenters told participants that they could leave the study at any time. In the laboratory session, participants were also informed in writing that the study requires audio and video recordings. By participating in the laboratory part of the study, the participants consented to these recordings; participants were also informed in writing that these recordings will be analysed and used in such a way that maintains anonymity for the participants.

We adhered to the APA standards of the time of data collection (2011) regarding conducting such experiments. By filling out a checklist from the Institutional Review Board of the University of Zurich we were informed that we did not need to seek formal ethical approval given that data in our study was collected anonymously and there were no potential ethical risks.

### Procedure

The teams—whose members did not know each other—were assigned randomly to either work on math problems or on EI problems. At the beginning of the experimental session, participants worked for ten minutes individually on items that were of the same type as the items in the upcoming team task. These were either 21 math tasks of varying difficulty from the Graduate Management Aptitude Test (GMAT, e.g., [64]) or 21 Emotional Intelligence items taken from self-scoring EI tests [65] and the MSCEIT [66]. Math items were scored according to the respective instructions and the EI items were scored with the consensus method. EI tests frequently employ consensus scoring where an item response is scored with the proportion of participants from a referent population who chose the particular item [67]. For this study, we created a referent sample consisting of 912 individuals that were drawn from the same population as the study participants. The proportion of item scores from this referent sample were used for scoring the EI items in the current study. None of the participants managed to complete all math or EI problems within the time limit. This individual task enabled us to determine participants’ actual task competence prior to the team interaction.

After participants familiarized themselves with the task in this way, we asked them to estimate the task competence of all other team members with a single item (“How competent do you think team member X is with regard to the task?”; [68, 69]) on a scale from 0–100. Subsequently, the team worked together on a single booklet containing further problems of the same task type for 30 minutes. This interaction was recorded on video, and these videos were coded with the Discussion Coding System (DCS, [70]) as we describe below. After the team interaction, participants provided demographic information on a final questionnaire, were debriefed, thanked, paid, and dismissed.

### Diversity beliefs manipulation

Prior to the team task but after the competence attributions, participants received a leaflet with information about the purpose of the study. This material was employed to manipulate participants’ diversity beliefs (see [8] for a similar manipulation). In the pro-similarity condition, participants learned that prior research has shown that gender-homogeneous teams tend to perform better on the task at hand and that members of gender-homogeneous teams find working with each other on such tasks more enjoyable than members of gender-heterogeneous teams. In the pro-diversity condition, participants were told the same with reference to gender-heterogeneous teams. The texts for both conditions referenced studies that supposedly reported the described findings. We told the participants in the debriefing that neither of the two simplistic statements is correct. As part of the debriefing procedure, participants received a leaflet summarizing the current state of research on gender diversity in teams, which contained references to current meta-analyses (e.g., [6]).

### Measures

#### Accuracy of competence attributions

Each team member was asked to rate the task competence of the three fellow team members at the outset of the team task. We determined the attributed task competence of each individual by averaging those three assessments. We tested whether averaging was permissible by calculating the average deviation around the mean (ADm; [71]) across the three ratings that each participant received from their team members (we thank an anonymous reviewer for this suggestion). Across all 388 participants, average ADm was 10.70. In other words, on the 100-point scale that we used, raters differed on average by around 11 points around the mean rating of attributed competence. Given a recommended threshold for aggregation of A/6 where A denotes the number of response options [72], average ADm was clearly below the applicable threshold of 100/6 = 16.66, with 84% of participants meeting the threshold, thereby justifying aggregation. We subsequently rank-ordered these individual-level attributions of task competence within each team, which resulted in a team-level hierarchy of attributed task competence.

To quantify the accuracy of this competence hierarchy, we correlated the within-team rank-order with the rank-order of members’ actual task ability by means of the *tau* non-parametric rank-order correlation coefficient. This resulted in a *tau* value between 1 for perfect alignment between task ability and attributed competence (thus indicating accurate competence perceptions in the team) and in -1 for a perfect negative association between the two, i.e., for a case where the member with the highest ability receives the lowest competence attribution. In the sample, observations of this team-level measure of the accuracy of competence attributions actually ranged from -1 to 1, *M* = 0.17, *SD* = 0.51. This variable was not normally distributed as indicated by a Shapiro-Wilk test, *W* = .95, *p* = .002. As a consequence, we employed robust techniques in the analyses involving this variable as we outline below.

### Elaboration of task-relevant information

We operationalized the elaboration of task-relevant information in the team as the number of speech acts that referred to the task content (i.e., the problem at hand) in a team’s interaction. These were identified by coding the video recordings of the teams’ interaction with the Discussion Coding System (DCS; [70]; see [41] for a previous use of the DCS to code elaboration). The DCS dissects the team interaction into individual statements or acts of communication according to a set of seven hierarchical rules. Each act is transcribed in brief. Based on the categories distinguished by Bales [73] and Fisch [74], the main function of a speech act is coded as belonging to one of three exclusive and extensive categories: An act can be a social-emotional statement (differentiated in positive or negative affect), a statement concerning the content of the task, or a statement aimed at regulating the discussion. In addition, for each speech act the DCS captures its accompanying interpersonal affect in terms of dominance or affiliation, its function (i.e., whether it is a question or a suggestion), and its responses in terms of agreement or rejection. It is thus adapted to the sequential, vertical, and reciprocal nature of interaction [75].

Two expert coders who were blind to the hypotheses and experimental conditions coded the videos with the DCS coding scheme in the video analysis software Mangold INTERACT [76]. Their agreement for the number of content speech acts was acceptable, Cohen’s *κ* = .68.

### Team member influence

Previous research operationalized team member influence with speaking time during a team interaction (e.g., [53, 77]). Accordingly, we operationalized the influence of team members on the elaboration process with their task-related speaking time. We obtained individual speaking time (in seconds) for all those speech acts classified as task-related from the INTERACT coding software [76] as part of the DCS coding process (see above). This approach resulted in an individual-level measure, which allows us to test Hypothesis 1 predicting that being perceived as competent and influential in the discussion are related.

### Team performance

In the team task section of the experiment, the team worked on 21 items of the same type as employed in the pretest. For a measure of team performance, we scored the solution to the problems that the team indicated on their joint answer sheet. Math items were scored according to the respective instructions and the EI items were scored with the consensus method. To compare the performance scores between teams working on math tasks and teams working on EI tasks, the scores were Z-transformed. None of the teams were able to complete all 21 items during the 30 minutes time limit, so no ceiling effects occurred in the performance variable.

### Manipulation checks

We checked the manipulation of diversity beliefs with four items in the post-task questionnaire: “Teams with members who are different from each other achieve higher performance than teams with members that are similar to each other”, “Teams with members who are different from each other experience more pleasant cooperation than gender-homogeneous teams”, “Teams with members from different genders achieve higher performance than gender-homogeneous teams”, and “Teams with members from different genders experience more pleasant cooperation than gender-homogeneous teams”. These items were presented with scales ranging from 1 (I strongly disagree) to 7 (I strongly agree).

### Control variables

We controlled for gender, age, individual’s task ability, and task type in the individual-level analysis for Hypothesis 1. For the team-level models, we controlled for team gender composition and task type. Team members’ task abilities were independent within teams, ICC(1) < 0.05, *p* > .20, and thus aggregation of individual ability to the team mean was not warranted. We employed the maximum ability (i.e., the individual ability score of the best team member) as a measure for team ability instead. Means, standard deviations, and bivariate correlations are given in Table 1 for individual-level variables, and in Table 2 for team-level variables.

**Table 1:** Individual-level means, standard deviations, and bivariate correlations of measurement variables and control variables.

	*M*	*SD*	1.	2.	3.	4.	5.	6.
1. Sex (female = 0, male = 1)								
2. Age	26.90	7.96	.19***					
3. Task (math = 0, EI = 1)			.01	.12*				
4. Diversity beliefs (0 = pro similarity, 1 = pro diversity)			.01	.05	.04			
5. Task ability (Z-transformed)^a^	-0.01	0.98	-.02	-.14**	.01	-.02		
6. Attributed competence	59.81	11.90	.04	.04	.21***	.01	.15**	
7. Influence (speaking time in s)	225.59	123.12	.14**	.11*	.26***	.05	.18***	.22***

*N* = 384 participants.

*p < .05

** *p* < .01

*** *p* < .001 (two-tailed)

*Note*. We refrain from reporting means and standard deviations for nominal factor variables.

^a^ We z-transformed math- and EI-scores independently before combining them into one overall performance variable, which is why the mean deviates from 0 and the standard deviation deviates from 1

**Table 2:** Group-level means, standard deviations, and bivariate correlations of measurement variables and control variables.

	*M*	*SD*	1.	2.	3.	4.	5.	6.	7.
1. Gender composition (2 men, 2 women = 0, 1 man, 3 women = 1)									
2. Gender composition (2 men, 2 women = 0, 1 woman, 3 men = 1)			-.50***						
3. Task (math = 0, EI = 1)			-.06	-.01					
4. Diversity beliefs (0 = pro similarity, 1 = pro diversity)			.03	-.01	-.04				
5. Team ability	1.01	0.84	-.07	-.03	.04	.04			
6. Competence accuracy	0.17	0.51	.01	-.17	.04	.10	-.07		
7. Elaboration	201.73	51.33	-.22*	.05	.58***	-.09	.01	.01	
8. Performance (Z-transformed)^a^	-0.03	0.97	.08	-.16	.03	.13	.12	.04	-.02

*N* = 94 groups.

*p < .05

** *p* < .01

*** *p* < .001 (two-tailed)

*Note*. We refrain from reporting means and standard deviations for nominal factor variables.

^a^ We z-transformed math- and EI-scores independently before combining them into one overall performance variable, which is why the mean deviates from 0 and the standard deviation deviates from 1

## Results

The raw data and analyses scripts are available online. We checked the data for extreme outliers with a multivariate outlier analysis [78]. Based on this analysis, we excluded a single team from the final data set employed in the analysis; the above bivariate correlation matrices contain the correlations for the final sample.

### Manipulation checks

In the pro-diversity condition, participants evaluated general team diversity as better for team performance (*M* = 5.34, *SD* = 1.24) and as more pleasant (*M* = 4.77, *SD* = 1.39) than in the pro-similarity condition (*M* = 3.55, *SD* = 1.62, and *M* = 2.91, *SD* = 1.26, respectively, *t*(358.58) = 12.20, *p* < .001, *d* = 1.24, and *t*(386) = 13.84, *p* < .001, *d* = 1.41). Likewise, participants in the pro-diversity condition evaluated gender diversity as better for performance (*M* = 5.62, *SD* = 1.24) and as more pleasant (*M* = 5.35, *SD* = 1.38) than in the pro-similarity condition (*M* = 3.52, *SD* = 1.66, and *M* = 3.67, *SD* = 1.59, *t*(383.42) = 14.06, *p* < .001, *d* = 1.43, and *t*(376.26) = 11.09, *p* < .001, *d* = 1.13). We thus deemed the manipulation a success.

### Attributed competence and influence on the elaboration process

To test whether team members who were perceived as being competent had more influence than those who were not perceived as being competent (Hypothesis 1), we regressed team member influence on their attributed competence, while controlling for their gender, their age, their task ability, the task type, and the diversity beliefs manipulation. Team member influence was independent of team membership, ICC1 < .01. In other words, measures of influence were not nested in teams, which renders the use of mixed models (i.e., hierarchical linear modeling or random coefficient models) unnecessary. Therefore, we used a regular regression model for testing the hypothesis. As shown in Table 3, the average female participant in the math task and in the pro-similarity condition elaborated for about three minutes (180.88 s). If we look at a male participant instead with all else being equal, men’s influence was about 28s larger, *SE* = 12.14, *p* < .05. Older participants also tended to have more influence, with each year increasing the influence by 1.3s, *SE* = 0.77, *p* < .10. Regardless of their gender and age, members spoke longer in teams working on EI problems (see Table 2). Pro-diversity beliefs had no significant effect on influence. Further, higher levels of task ability affected the influence of team members such that a level of individual ability one standard deviation above the sample mean resulted in about 22s more influence, *SE* = 6.18, *p* < .001. Finally, in support of Hypothesis 1, competence attributions affected team member influence: An increase of one unit of attributed competence (on the scale from 0–100) resulted in 1.42s more influence, *SE* = 0.52, *p* < .01. Given that the difference between the highest and lowest score of attributed competence in our sample was 65 points, this model predicts that the influence of the participant with the highest attributed competence was about 92s larger than that of the participant with the lowest level of attributed competence. Against the backdrop of about three minutes of average influence, attributed competence, controlled for task ability, therefore had a significant impact on team members’ influence on the elaboration process. As a further robustness check, we re-ran this model as a random-intercept multilevel model. It revealed the same finding: attributed competence significantly affected influence, gamma = 1.42, SE = 0.52, t = 2.75, 95% CI = [0.41; 2.43]. The fact that the confidence interval of the random effect variance of the intercept included 0, 95% CI = [0.00; 21.95] further illustrates the equality between the random intercept model and the OLS regression reported in Table 3.

**Table 3:** Individual-level regression of influence (speaking time in seconds) on study variables.

	*b*	*SE*	*t*
(Intercept)	180.88	11.89	15.210***
1. Sex (female = 0, male = 1)	28.05	12.14	2.311*
2. Age (mean-centered)	1.31	0.77	1.696^†^
3. Task (math = 0, EI = 1)	52.79	12.29	4.294***
4. Diversity beliefs (0 = pro similarity, 1 = pro diversity)	-8.91	11.93	-0.746
5. Task ability (Z-transformed)	21.66	6.18	3.506***
6. Attributed competence (mean-centered)	1.42	0.52	2.745**

*N* = 384 participants. Adj. *R*^*2*^ = .13, *F*(6,366) = 10.27, *p* < .001.

^†^*p <* .*10*

*p < .05

** *p* < .01

*** *p* < .001 (two-tailed)

### Accuracy of competence attributions, elaboration, team performance, and diversity beliefs

Hypothesis 2 predicted that the relationship between information elaboration and team performance is moderated by the accuracy of the competence attributions, such that elaboration is detrimental in the case of inaccurate competence perceptions and beneficial when competence attributions are accurate. To test this hypothesis, we conducted a stepwise regression of team performance on information elaboration, accuracy of competence attributions, and their interaction. The first step regressed team performance on team gender composition, task type, team ability, the diversity beliefs manipulation, and the main effects of elaboration and the accuracy of the competence attributions. However, regression diagnostics revealed that the residuals were not normally distributed, but heavily tailed due to the fact that the competence attribution accuracy variable was not normally distributed (see above). The assumption of independently and normally distributed residuals was thus violated. We therefore tested the proposed relationships with robust regressions [79], which can deal with such violations by attributing less influence to observations that distort the overall result. Specifically, we employed the rlm() function provided in the MASS package [80] of the statistical envirionment R [81]. Following the suggestions by Venables and Ripley [80], we used 95% confidence intervals based on non-parametric bootstrapping for significance testing because these do not require distributional assumptions for statistical inference, see Table 4, Step 1.

**Table 4:** Robust regression of group performance on study variables.

	Step 1			Step 2			Step 3		
	*b*	*LL*	*UL*	*b*	*LL*	*UL*	*b*	*LL*	*UL*
(Intercept)	-0.122	-0.502	0.283	-0.115	-0.503	0.279	0.167	-0.260	0.654
Gender composition (balanced = 0, 1 man, 3 women = 1)	-0.136	-0.562	0.441	-0.015	-0.550	0.517	0.033	-0.492	0.550
Gender composition (balanced = 0, 1 woman, 3 men = 1)	-0.365	-0.808	0.050	-0.387	-0.781	0.037	-0.412	-0.816	0.013
Task (math = 0, EI = 1)	0.015	-0.420	0.479	-0.041	-0.515	0.381	-0.109	-0.605	0.328
Diversity beliefs (0 = pro diversity, 1 = pro similarity)	-0.307	-0.710	0.106	-0.289	-0.679	0.129	-0.268	0.686	0.085
Team ability (mean-centered)	0.178	-0.126	0.455	0.151	-0.182	0.412	0.083	-0.266	0.381
Competence attribution accuracy (mean-centered)	0.032	-0.303	0.431	-0.003	-0.317	0.361	0.235	-0.191	0.762
Elaboration (mean-centered)	-0.002	-0.006	0.003	-0.001	-0.005	0.003	-0.004	-0.009	0.004
C × E				0.007	0.001	0.013	0.010	0.002	0.022
C × DB							-0.463	-1.246	0.218
DB × E							0.005	-0.003	0.013
C × E × DB							-0.003	-0.019	0.008

*N* = 97 groups. *b* = unstandardized regression weight obtained through non-parametric bootstrapping, *LL* = 95% CI lower limit, *UL* = 95% CI upper limit. Confidence intervals were calculated using the adjusted bootstrap percentile (BCa).

**Fig 1:**
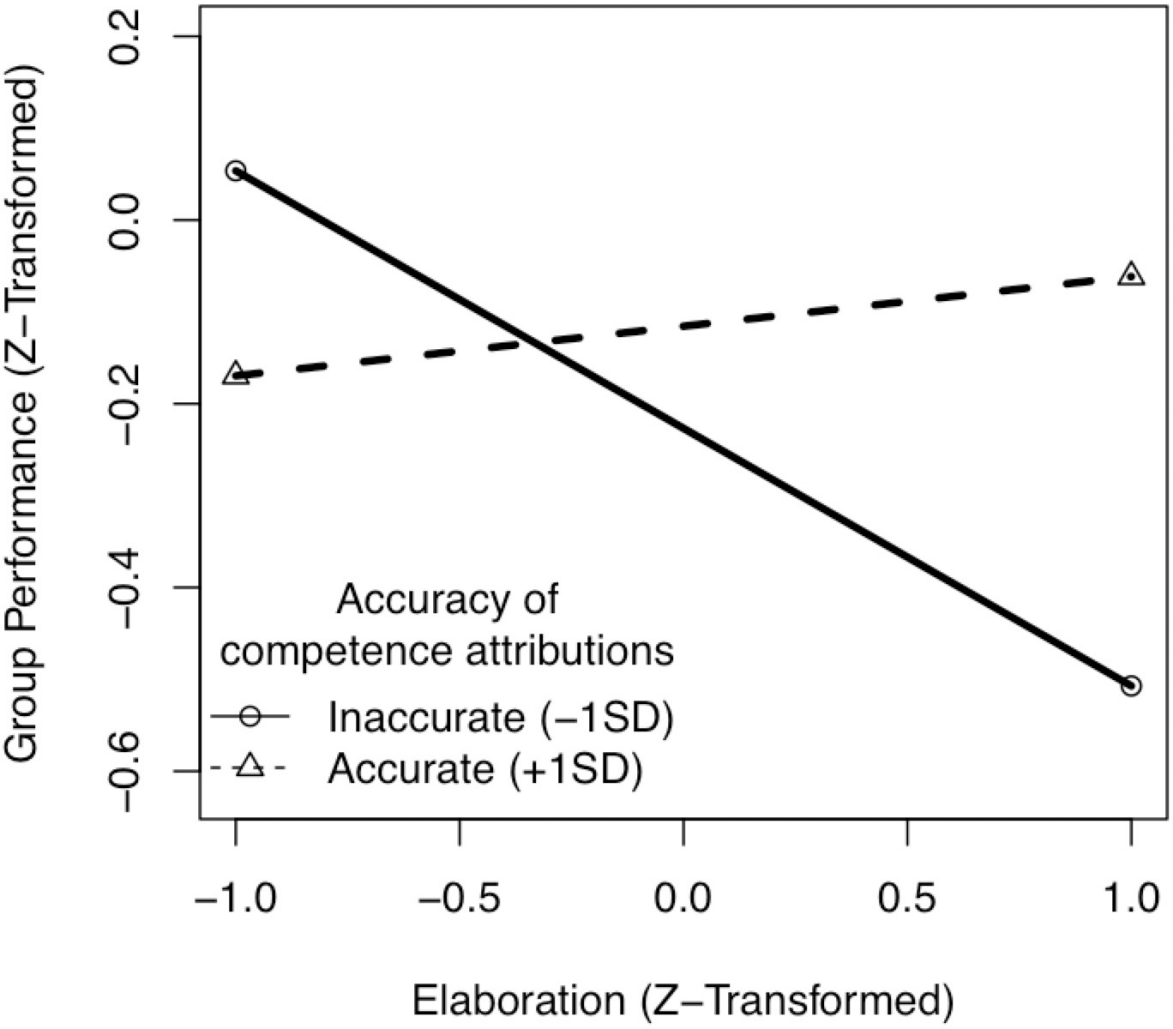
Interaction between elaboration and the accuracy of competence attributions on group performance.

In the second step we added the interaction between competence attribution accuracy and information elaboraton to the previous regression [82]. Step 2 in Table 4 revealed a significant interaction between competence attribution accuracy and information elaboration. To foster its interpretation, we plotted the interaction following the conventions by Aiken and West [83]. i.e., we plotted the relationship between the focal predictor, elaboration, and the dependent variable, group performance at two different levels of the moderator: Accuracy of competence attribution one standard deviation below its mean (inaccurate attributions) and one standard deviation above its mean (accurate attributions) with the tools provided by Preacher, Curran and Bauer [84], see Fig 1.

In support of Hypothesis 2, an increase in the level of elaboration led to a decrease in performance in teams with inaccurate competence attributions: The simple slope of elaboration for teams with inaccurate competence attributions was -0.28 (*SE* = 0.14), *t* = -1.98, *p* = .05. For teams with accurate competence attributions, the relationship between elaboration and performance was positive yet nonsignificant (simple slope = 0.05, *SE* = 0.15, *t* < 1). Neither task type, nor the gender composition of the team, nor their interaction influenced the team’s performance, and controlling for these variables did not make the interaction of competence attribution accuracy ×<elaboration disappear.

Given that the convention of plotting interactions at one standard deviation above and below the mean of the moderator is somewhat arbitrary, we also determined the region of significance for the interaction [84]. It revealed a negative relationship between elaboration and group performance that reached significance for z-transformed accuracy levels below -1.01. In other words, elaboration hurt performance in groups with quite inaccurate status attributions and did not affect performance in groups with more accurate attributions.

In rejection of Hypothesis 3, the three-way interaction between elaboration, competence attribution accuracy, and the diversity beliefs manipulation turned out to be not significant (see Step 3 in Table 4).

## Discussion

Our finding that information elaboration can decrease a gender-diverse team’s performance opposes conventional wisdom in theory and research on the relationship between team diversity and team performance. In a field that has been confronted with many inconclusive findings [6], the idea that information elaboration enhances the performance of diverse teams has been one of the very few strongholds. In fact, the concept of information elaboration has become so popular that even in the wider literature on teams (i.e. beyond the scope of diversity), information elaboration has been considered as a driver of team performance (e.g., [26, 10]). Given that scientific progress depends on existing ideas, theories and assumptions being challenged and tested [85], we assert that our qualification of the benefit of information elaboration provides an important impetus to the field. A particular strong point that bolsters the robustness of this finding is that we relied on behavioral observations and objective performance data, which enabled us to show that the detrimental effects of information elaboration in teams with inaccurate competence attributions were due to the high influence of the members who were inaccurately perceived as competent. Our finding that pro-diversity beliefs did not mitigate the negative effect of information elaboration on performance for teams with inaccurate competence attributions, albeit unexpected, lends further credence to the robustness of our finding that information elaboration can negatively affect team performance.

### Theoretical implications and directions for future research

Our study advances theory and research on information elaboration in diverse teams in several ways. First, we build theory on the process of information elaboration by arguing and showing that members who are perceived as more competent (while controlling for task ability), are more influential in the information elaboration process. This is an important finding, given that ample theory and research shows that competence is attributed automatically during the first moments that teams are formed [19, 20, 50, 63, 73]–especially when members differ from each other in terms of characteristics that are believed to relate to competence on the task at hand [86]. In line with the recent coordination perspective on diversity [13, 14], our findings thus suggest that especially in diverse teams information elaboration is likely to be a disparate process in which some members are more influential than other members. Because gender stereotypically is thought to be related to performance on math or EI tasks, for the current experiment we used gender to create differences in competence attributions among team members. For other task contexts, there may however be cues other than gender that affect attributions of competence (e.g., tenure, [20]; functional background, [87]). We thus extend theory on whether and how information is processed and elaborated in diverse teams by pointing at the role of context and attributions of competence in shaping disparities in member influence in the information elaboration process. It should be noted that we only showed this in a context where gender diversity matters, and that future research should assess to what extent these insights also hold for other types of diversity in other task contexts. However, although competence attributions based on job-related types of diversity are more likely to be accurate than attributions of competence based on gender or other demographic diversity types, we expect that the manner in which such competence attributions will shape disparities in member influence in the information elaboration process will be the same.

Second, we argued and showed that such attributions of competence are highly consequential for the outcomes of the information elaboration process: when attributions of competence are inaccurate, information elaboration harms performance. In a field where information elaboration is considered to be a silver bullet (e.g., [8, 9, 11]), this is a worrisome conclusion. Indeed, although recent studies indicate that information elaboration may not always benefit teams (12, 26]), these studies do not indicate that information elaboration can actually harm performance. Our theory and findings are however congruent with research on groupthink [88] by indicating that more discussion is not always a good thing and, in fact, can lead a team further astray. Moreover, groupthink research indicates that teams are more likely to make poor decisions when the person who is most influential (e.g., the person who is attributed highest competence, or the leader) is not the most competent team member [89], which aligns well with our findings. Although our study thus opposes conventional wisdom in diversity research, it aligns well with other literature on team functioning.

Somewhat in contrast to our expectations, more elaboration in teams with accurate competence attributions did not enhance performance. At least in part, this may be a power issue, given that the number of teams in our sample was limited and the relationship between information elaboration and performance for teams with accurate competence attributions was nonsignificant, yet positive. However, in light of the findings of Resick et al. [26], another reason for this finding may have to do with the type of task employed in our study. Specifically, in some other studies where information elaboration was found to increase performance, participants worked on hidden profile tasks that require members to exchange and integrate unique pieces of information that are held by different members (e.g., [10]). The tasks in our study are more disjunctive [90]: whereas for some of the items an exchange and integration of insights may have helped a team to reach the correct solution, for most items it is likely that whether or not a team found the correct answer depended on the task ability of a single team member. For such tasks, information elaboration might be less critical. As a consequence, a third contribution of our study is that it complements the findings of Resick et al. [26] by suggesting that information elaboration may not enhance performance for disjunctive tasks where finding the correct or right solution depends more on the competence of a single team member.

Although we thus did not find that information elaboration enhances performance when attributions of competence are accurate, our study does indicate that it is important to have accurate competence attributions for information elaboration not to harm performance. We therefore call for research on factors that contribute to (increasing the ability of team members to improve) the accuracy of competence attributions. In this regard, it is interesting to note that in more than one-third of our teams the members who were the most competent did not fit the stereotype (i.e. women were better in math and/or men were better in EI). Gaining accurate competence attributions thus requires team members to look beyond their initial stereotypes and impressions. As a consequence, every factor that enhances the collection of individuating information is likely to be beneficial (cf. [14]). We therefore recommend future research to examine whether well-known individuation-enhancing factors such as intergroup contact [91, 92] and low levels of acceptance of stereotypes [93] yield more accurate competence attributions.

Obtaining accurate attributions of competence may however not always be possible–for example because there is no time for getting to know one another, or because people may pretend to be more competent than they are. How can such teams that may suffer from inaccurate competence attributions minimize the negative consequences of such inaccurate competence attributions? To address this question, our fourth contribution consisted of exploring whether pro-diversity beliefs are helpful here. Because pro-diversity beliefs are known to make ingroup members more open for input from outgroup members [8, 35], we anticipated that pro-diversity beliefs might also make members more receptive for input from members whom are attributed low competence. We can only speculate about the reasons why this hypothesis was not supported, but there are at least two lessons to be learned here. First, even though in our sample attributions of competence to a large extent were based on gender and diversity beliefs are known to affect how members of different genders interact with each other; diversity beliefs do not seem to affect how members interact with members of different levels of attributed competence. Pro-diversity beliefs thus inhibit the effects of social categorization and intergroup biases on interaction and elaboration, but not the effects of attributions of competence on interaction and elaboration–even though those attributions of competence are at least in part based on social categorization and intergroup biases. Second, because pro-diversity beliefs in other studies have consistently shown to positively affect performance in diverse teams (for a meta-analysis, see [94]), the finding that pro-diversity beliefs do not mitigate the negative effect of information elaboration on performance when competence is inaccurately attributed in teams speaks to the robustness of the effect of inaccurate competence attributions and puts a premium on research that can help teams to overcome the detrimental effects of inaccurate competence attributions. Arguably, any practice aimed at reducing the influence of team members with high levels of attributed competence and increasing the participation of team members with low levels of attributed competence may be helpful, including a leadership style that facilitates open communication and trust in diverse teams, such as transformational leadership [95]. However, the difficulty here is that members of teams with inaccurate competence attributions generally are not aware of the inaccuracy of their beliefs about their team members’ task ability. We therefore believe that the safest way to overcome the negative consequences of inaccurate competence attributions lies in gathering individuating information that enables team members to obtain a more accurate understanding of the competence levels of their fellow team members.

### Strengths and limitations

Strengths of our study include the reliance on behavioral observation for assessing the level of influence of members on the information elaboration process as well as for measuring information elaboration. Further, we used objective performance indicators, which in diversity research is clearly preferred above subjective performance indicators [6]. Another quality of our study is that our dataset includes two different task contexts. Whereas the math task corresponds with settings where teams can primarily rely on logic, for emotional intelligence a different skillset is needed. The fact that no difference in effects was found between the two task contexts strengthens the external validity of our study.

That is however not to say that our study is without limitations. Our study has been conducted with newly formed teams where team members do not know each other. This raises the question to what extent our findings hold in teams where members have a history of working together and know each other well. Whereas the source of competence attributions may change over time [20], we believe there is no reason to expect that the effect of the accuracy of competence attributions on the relationship between information elaboration and performance will differ. However, it would be good to validate this claim by replicating this study in an organizational sample with teams where members have a long history of working together.

A related issue is that our study was conducted in an artificial setting with students as participants. To mirror reality, students had to work on real tasks for which they received either ‘participation credits’ (compulsory for psychology students), or a financial reward Furthermore, a meta-analysis [6] did not find any meaningful difference in the relationship between diversity and team performance between studies conducted in a lab versus in the field. Nevertheless, also for this reason it would be good to replicate this study in an organizational sample.

Longitudinal research is needed to examine whether attributions of competence become more accurate over time. Intergroup contact theory, for example, suggests that members gather more individuating information and hence a more accurate understanding of other team members’ competence over time because team members have more information to estimate another’s expertise on the task at hand [91, 96]. However, research on the reinforcing nature of status (e.g., status hierarchies theory, e.g., [51, 97]; stereotype maintenance theory, e.g., [98]; prescription-based discrimination, e.g., [22]) suggests that initial impressions may set the stage for any subsequent impression of team members about their task ability (for a recent overview of these contrasting perspectives, see [99]). Indeed, the tendency for high-status team members to gain more influence the more teams engaged in information elaboration provides some evidence for the latter theory, but our study clearly does not test this.

### Managerial implications

Our study speaks to the value of information elaboration in diverse teams in a way that has not been done before: Information elaboration can be detrimental when attributions of competence are inaccurate. In contrast to what has been suggested in other studies [8, 10, 11], a clear implication of our study thus is that practitioners should not take the positive effects of information elaboration in diverse teams for granted. Specifically, our study shows that when information elaboration negatively affects performance, this is due to an increased influence of team members who are inaccurately perceived as competent in the information elaboration process. It is easy to see how often this can happen in a real life situation. More often than not, task allocation is based on attributed compentence rather than actual compentence or preference for the task [100].

Of course, the immediate question that follows is how managers and colleagues can enhance the accuracy of competence attributions. Unfortunately, our paper stays mute to that question. However, as indicated above, individuation–the process of looking beyond social categories, perceiving each individual as an individual and acknowledging everyone’s ideosyncracies–should enable employees to more accurately estimate a person’s true abilities [14]. In addition, managers would do well to inventarise how competence is attributed in their teams/organization. Competence is frequently attributed based on superficial aspects like a member’s characteristics [17, 86]–factors that might have nothing to do with a member’s actual competence. The more that members rely on such superficial aspects and stereotypical beliefs to attribute competence, the more likely it is that competence attributions are inaccurate and cause information elaboration to negatively affect team performance.

### Conclusion

In the present study we challenge the notion that information elaboration is the key to unlocking the potential of (diverse) teams. In line with the recently advanced coordination perspective on diverse teams [13, 14], our findings indicate that information elaboration in diverse teams tends to be a disparate process in which members are more influential when they are attributed higher levels of competence. Because information elaboration is shown to harm performance when such attributions of competence are inaccurate and the most influential team members thus are not those with the highest levels of task ability, we put a premium on future research efforts that identify how the accuracy of competence attributions in diverse teams can be enhanced.
